# Generation of stable advective-diffusive chemokine gradients in a three-dimensional hydrogel

**DOI:** 10.1063/5.0064947

**Published:** 2022-02-16

**Authors:** Willy V. Bonneuil, Daniel J. Watson, Jennifer Frattolin, Matthew J. Russell, Francesca Fasanella Masci, Mikaila Bandara, Bindi S. Brook, Robert J. B. Nibbs, James E. Moore

**Affiliations:** 1 Department of Bioengineering, Imperial College London, London SW7 2BP, United Kingdom; 2 School of Mathematical Sciences, University of Nottingham, Nottingham NG7 2RD, United Kingdom; 3 School of Life Sciences, University of Glasgow, Glasgow G12 8TA, United Kingdom

## Abstract

Physiologic chemoattractant gradients are shaped by diffusion, advection, binding to an extracellular matrix, and removal by cells. Previous *in vitro* tools for studying these gradients and the cellular migratory response have required cells to be constrained to a 2D substrate or embedded in a gel devoid of fluid flow. Cell migration in fluid flow has been quantified in the absence of chemoattractant gradients and shown to be responsive to them, but there is a need for tools to investigate the synergistic, or antagonistic, effects of gradients and flow. We present a microfluidic chip in which we generated precisely controlled gradients of the chemokine CCL19 under advective-diffusive conditions. Using torque-actuated membranes situated between a gel region and the chip outlet, the resistance of fluid channels adjacent to the gel region could be modified, creating a controllable pressure difference across the gel at a resolution inferior to 10 Pa. Constant supply and removal of chemokine on either side of the chip facilitated the formation of stable gradients at Péclet numbers between −10 and +10 in a collagen type I hydrogel. The resulting interstitial flow was steady within 0.05 *μ*m s^−1^ for at least 8 h and varied by less than 0.05 *μ*m s^−1^ along the gel region. This method advances the physiologic relevance of the study of the formation and maintenance of molecular gradients and cell migration, which will improve the understanding of *in vivo* observations.

## Introduction

Chemoattractants are produced by multiple cell types distributed in 3D *in vivo*. They diffuse, are advected, and bind to extracellular matrix (ECM) or cell surface molecules to form functional gradients that guide cell migration.^1^ Advection shapes soluble gradients, affects the distance along which molecules can be transported before binding to receptors or ECM,^2^ and enables autologous chemotaxis.^3^ The Péclet number, which represents the ratio between advective and diffusive transport fluxes, may be in the diffusiondominated range (<1), in the advection-dominated range (>1), or in the transitional range (~1) *in vivo*, so it is important for *in vitro* models to provide fine and dynamic advection control to encompass all of these situations. Crucially, several cell types, including tumor cells,^4–6^ fibroblasts, and smooth muscle cells,^7^ were shown to adjust their migration in response to flow only. It is thus also important for *in vitro* models to have the capacity to separate the roles of flow and concentration gradients in guiding cell migration.

Research on cell migration in response to chemotactic gradients *in vitro* has advanced by using both 2D substrates and 3D environments, incorporating e.g., ECM-derived hydrogels. 3D environments allow cells to assume physiologic morphologies and permit a more faithful modulation of the signaling cues that regulate their function, possibly by obviating the need for adhesive ligands.^8^ Microfluidic chambers can be designed for either 2D or 3D cell culture and provide the possibility to apply chemotactic gradients in various ways. Some designs used laminar-flow patterning to produce soluble gradients over the apical surface of 2D cell cultures,^9–12^ while others employed diffusion from a supply channel into a cell-laden hydrogel.^13–15^ Solid-phase gradients have also been established by binding chemokines in graded fashion onto a glass substrate.^16,17^ Each of these techniques has limited capability to reproduce the relevant 3D *in vivo* conditions under which cells migrate chemotactically or haptotactically. Laminar-flow patterning can only produce soluble chemoattractant gradients parallel to the cell surface and perpendicular to the flow direction. Gradients of arbitrary orientations were demonstrated in devices based on these techniques,^11^ but the direction and magnitude of the accompanying fluid flow are determined by chip design rather than specific physiologic goals. Furthermore, the high fluid velocities required to establish the gradients may also cause the gradients experienced by the cells to deviate significantly from the desired ones.^18^ A recent device extended the principle of laminar-flow patterning to 3D hydrogels, and the fluid velocities it requires are low enough so as not to cause this deviation.^19^ However, the molecular gradients are still constrained to be orthogonal to fluid flow and, as the authors note, the gradients depend on the position along the fluid streams, which renders statistical analysis of cell migration more complex. Binding chemoattractants to substrates exposes controllable haptokinetic gradients to the basal sides of adhered cells but is similarly limited to two dimensions, both in terms of the gradients and the cell culture. In microfluidic chambers that employ fluid barriers, such as an agarose barrier^20^ or a porous membrane,^21^ stable and controllable diffusive gradients allow the observation of cell migration under more realistic 3D conditions. However, their designs eliminate fluid flow through the cell-containing matrix. Hence, these designs result in three limitations. First, the gradients are limited to diffusion-dominated transient or linear steady-state profiles. Second, these designs do not allow the study of fluid flow effects that are known to directly affect the migration of cells even in the absence of external gradients. Third, they do not allow migrating cells to form the potentially important autologous gradients^22^ that they can secrete in the absence of external chemical signals when subjected to flow.^3,23^

The control of interstitial flow (IF) across gel regions within microfluidic chambers most often involves either open reservoirs for gravity-fed pressure-driven flows^24–27^ or peristaltic pumps that behave as nearly ideal flow sources.^28–31^ Reviews of these and other methods, including droplet-based methods, can be found in Refs. 32 and 33. Pressure-driven setups can provide relatively stable fluid velocities, but their precision is limited by the degree to which the fluid height difference can be maintained constant. Peristaltic pumps create a non-physiologic flow pulsatility that is typically dampened with compliance chambers. However, these require a significant dead volume of fluid that potentially contains expensive ingredients. Syringe pumps can provide reliably steady flow, but the pressure within the chip depends very sensitively on the downstream resistances. Thus, the problem of consistently generating a reliable pressure difference across a hydrogel is shared by both ideal pressure and ideal flow sources.

Some studies have investigated the combined effects of gradients of Vascular Endothelial Growth Factor (VEGF) and IF in 3D matrices. These chambers contained a central gel-containing observation region framed by two parallel fluid channels fed by reservoirs of different heights. The capacity of these devices to adjust flow on-the-chip was limited to a resolution of 10 Pa in one of these studies, which corresponds to 10 Péclet-units for VEGF.^25^ This means that gradients can be either diffusion-dominated, or advection-dominated in multiples of 10 only. Furthermore, boundary concentrations were not actively maintained, which led to flat concentration profiles at high Péclet numbers. Finer control of the Péclet number was achieved in another study by adjusting the width of the gel region in the chips, which required new devices to generate different experimental conditions.^26^

Solving the problem of controlling advective-diffusive gradients across hydrogels requires strategies that provide sufficiently precise and accurate control over the pressure distributions and the chemo-attractant concentrations on either side of the gels. First, we aimed to have sufficiently precise control over the pressure differences across a gel region such that we could generate advection velocities of physiologic magnitudes for interstitial fluid. This control should have a resolution of 0.1 *μ*m s^−1^ to finely cover situations close to the average homeostatic interstitial velocity of 0.5 *μ*m s^−1^.^34^ Second, we aimed for the advection to be constant without needing external action, so that it would generate constant gradient shapes. Third, we aimed to limit the variation in advection along the gel region. Generating uniform advection and chemo-attractant concentration fields would allow observations of gradient formation and cell response, in future work, to be independent from their location within the chip. Finally, we aimed to be able to modulate the level and direction of advection (enhancing or opposing diffusion) without disrupting the experimental setup. This would allow concentration profiles to be changed dynamically, as happens *in vivo*, and isolate chemo-attractants that have bound to matrix components by washing out their soluble forms.

There are several designs of microfluidic chambers that allow a pressure differential to be applied across a central observation region. The latter can be defined by circular posts^29^ or by microgrooves,^35^ among others. Here, we pursued an adaptation of the specific geometry developed by Kamm’s group with trapezoidal posts caging a gel region^36,37^ and report the incorporation of screwactuated resistive membranes that allow advection across a collagen I gel to be controlled accurately at a precision of 0.1 *μ*m s^−1^ for periods of at least 8 h. We adapted the geometry of the chambers to limit the deviation of advection along the gel region and demonstrate the ability to modulate it and even change its direction on-the-chip. This is realized for the non-ECM-binding chemokine CCL19, which acts on CCR7–positive immune cells.^38,39^

## Methods

**Fig. 1:**
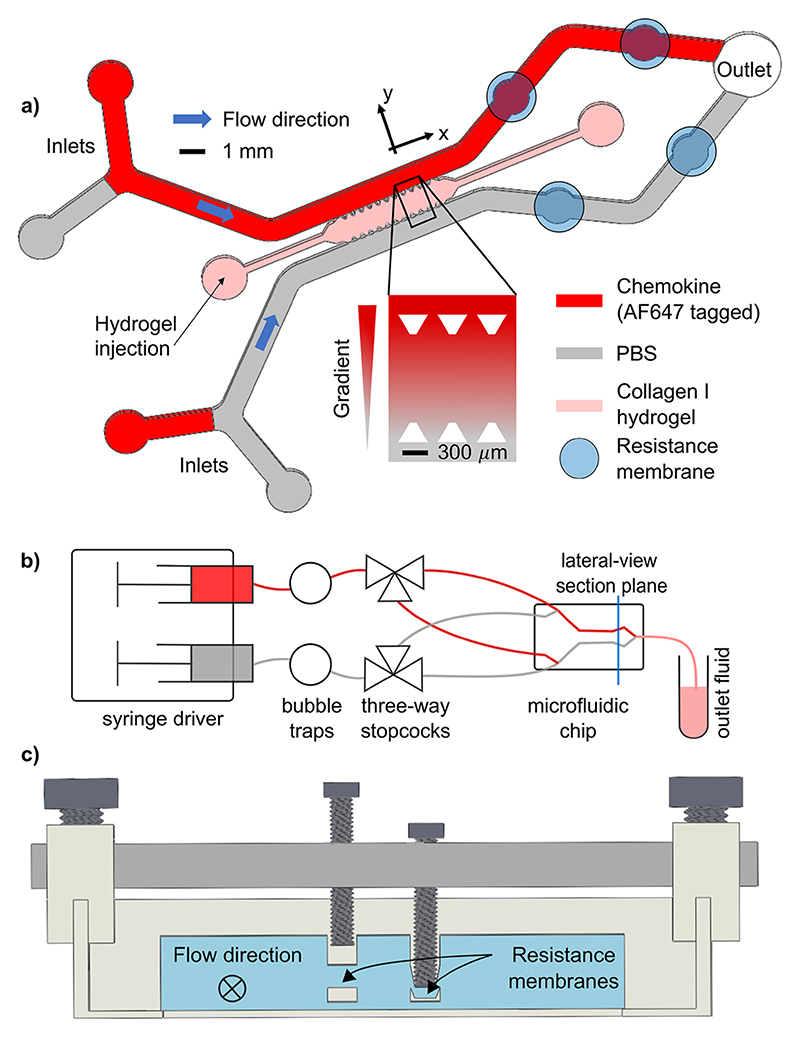
(a) Top-down view of the microfluidic circuit gel domain based on Ref. 36. Membranes placed between it and the outlet can be deflected to increase the upstream pressure. Inset: expansion of the gel domain. Trapezoidal posts keep the gel in place while allowing fluid flow into and out of it. (b) Overall flow circuit during gradient generation experiments. (c) Cross-sectional view from the plane of the resistance membranes. The fluid channels are higher and the resistance membranes are thicker relative to the rest of the setup than their actual dimensions.

Our objective was to generate controllable advection in a microfluidic chip containing a rectangular gel region, framed on two sides by fluid channels and separated from them by trapezoidal posts that cage the gel, while enabling the passage of macromolecules, cells, and fluid [Fig. 1(a)]. While the eventual goal is to include migrating cells within the gel, we focused this report on the microfluidic design work necessary to achieve controllable advection of chemo-attractants with precision, steadiness, uniformity, and onchip modularity. To attain these four design aims, we combined an experimental circuit and two mathematical models. A lumpedparameter model demonstrated the effects of microfluidic design and operation parameters on the generation of physiologic gradients and a 3D computational model guided our processing of experimental gradients. We previously used an earlier version of this chip and fluorescent imaging to quantify the diffusivity of CCL19 at physiologic concentrations (less than 10 nM^40^).

The pressure differences across the gel should generate advection velocities within the range of physiologic values for interstitial fluid (0.1–10 *μ*m s^−1 34,41^). For CCL19 whose diffusivity *D* is of the order of 130 *μ*m^2^ s^−1^, and a characteristic transport length *L* equal to the gel region width, 1.3 mm, the Péclet number at a velocity *v*,

$$Pe=\frac{vL}{D},$$

should be less than 100. However, concentration profiles for |Pe| > 10 would be almost completely flat across the gel. Therefore, we set a design criterion to achieve precise control over the Péclet number in the range of −10 to 10, corresponding to advection velocities of up to 1 *μ*m s^−1^ for CCL19.

### Microfluidic circuit operation and gradient observation

Screw-actuated resistance membranes were chosen to induce precise and finely adjustable pressure differences across the gel region. Their capacity to continuously vary the fluid-channel cross-section under them when deflected and to maintain this constriction indefinitely justified their use. The principle of microfluidic resistances was implemented in a few previous microfluidic studies. Resistances modulated with pneumatically deformable membranes to block flow channels that function more or less as on/off switches were incorporated into a stream-mixing device to skew gradient profiles dynamically.^42,43^ Others have used torque-actuated valves in stream-mixing^44^ and reagent delivery^45^ applications. They demonstrated that torque-actuated valves had equal performance to pressure-actuated valves while being able to remain deflected without external action and exhibiting a lower footprint and cost.

The microfluidic chips were molded into polydimethylsiloxane (PDMS) (Sylgard 184, Dow Corning, USA) from custom-made SU-8 wafers (FlowJEM, Canada). The fluid was supplied to the chip inlet by a common syringe driver (Cole-Parmer, USA) to which one syringe of chemokine and one of phosphate-buffered saline (PBS) were fitted. Each supply line contained stopcocks of low dead space (0.02 ml) (Kinesis, UK) that branched to two opposite pairs of fluid inlets [Fig. 1(b)]. This allowed the intermittent and rapid delivery of chemokines to both fluid channels in the chip and, therefore, to both sides of the gel region. A pair of M1.4-screw-actuated valves in a control layer was placed between the gel region and the outlet over both fluid channels [Fig. 1(c)]. The indentations guiding the screws were made by attaching 3D-printed racks with posts corresponding to the valve locations to the wafer prior to pouring uncured PDMS on it. These posts were designed to create a 200 *μ*m membrane between the bottom of the racks and the fluid channels that could be easily deflected by the screws. The screws were held in place by a 3-mm thick aluminum plate to prevent recoil, which was itself fastened to a 3D-printed chip holder. The fluid channel under the membranes had a circular wall profile to distribute strain more uniformly. The diameter of this space was 0.1 mm larger than the screw shaft in order to reduce strain in the PDMS and to make the adjustment of the resistance at high deflection less sensitive to small variations. The fluid channels met at a common outlet to ensure that any pressure difference between the two sides of the gel only arose from the screwguided deflections and not from differences in outlet surface tension or outlet tubing length.

Collagen I gels were prepared at a concentration of 2.0 mg ml^−1^ according to the collagen supplier’s specifications (Dow Corning, USA). Human CCL19 chemokine fluorescently labeled with Alexa Fluor 647 (AF647) (Almac, UK) was dissolved in PBS to a physiologic concentration of 10 nM,^46^ incubated at 37 °C for 30 min and vortexed for 1 min before use to ensure its complete dissolution. After the gelation, the fluid channels were primed with PBS from gas-tight syringes (Hamilton, USA). The gel ports were sealed with cyanoacrylate + sodium bicarbonate and the chip was pressurized by a 1-m PBS column to eliminate all bubble nucleation sites.^47^ The chips were imaged using a confocal microscope (SP8, Leica, Germany) at room temperature, under a 10× objective and with a pinhole aperture of 0.80 Airy. Photons were excited with a white light laser emitting at 633 nm at 400 mW and were counted with a hybrid detector gated between 0.3 and 4.5 ns. The images taken were strips of 1552 × 388 *μ*m^2^ containing the width of the gel region between two opposite fluid–gel interfaces, plus some of the adjacent fluid channels for concentration references. The corresponding resolution was 2048 × 512 pixels. Brightfield images were taken in parallel to help locate the fluid–gel interfaces and verify the integrity of the gel. The calibration of the resistance screws was done prior to each experiment by adjusting the screws until the deflection of the coverslip was detected through the microscope.

### Lumped-parameter modeling of fluid flow in the chip

**Fig. 2:**
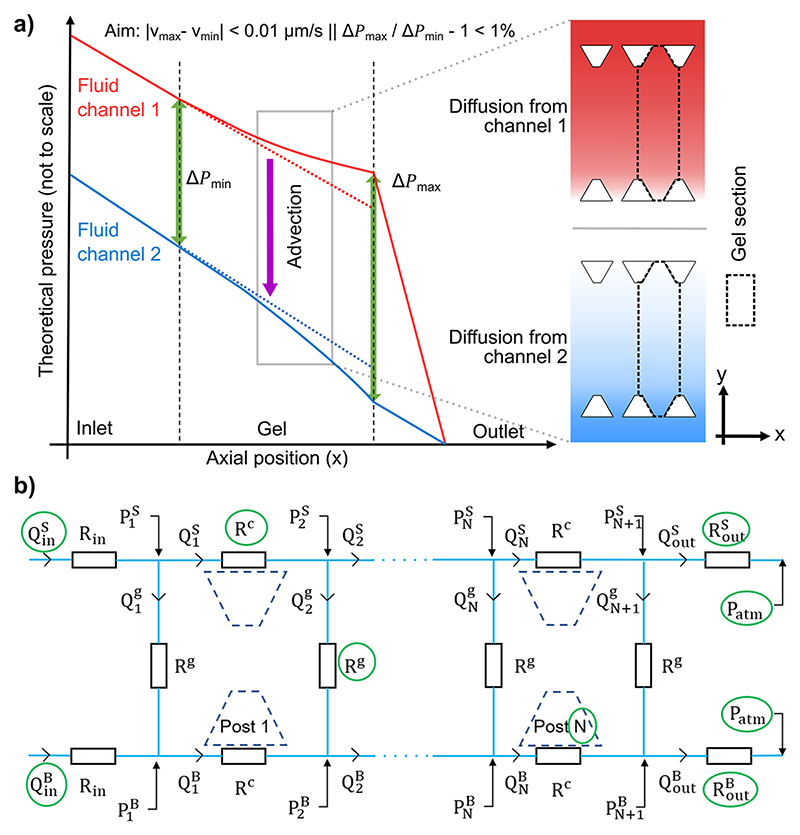
(a) Theoretical representation of pressure distributions in the fluid channels. Right insets: corresponding gradients in our chip. A concave gradient generated from a molecule diffusing from channel 1 (red) and a convex gradient from channel 2 (blue). A gel section is defined as the domain centered on the middle of opposite inter-post gaps and framed by the axes of symmetry of adjacent opposite post pairs. (b) Lumped-parameter representation of the chip used for its hydraulic modeling. In a chip with *N* posts, the gel domain is divided into *N* + 1 sections, which are assumed to separate the cross-gel flow. The green circles represent design parameters. *P* = pressure, *Q* = flow rate, and *R* = resistance. Subscripts or superscripts: *in* = inlet, *out* = outlet, *S* = source, *B* = buffer, 1 to *N* or *N* + 1 post or section number, *c* = fluid channel unit along one post, and *h* = gel.

To help ensure the uniformity of the pressure difference Δ*P* (and thus, advection) across the gel, we constructed a lumpedparameter model in which the chip design and experimental parameters could be varied. In the channel where the mean pressure is higher, fluid is lost to the gel region and the pressure gradient decreases along the *x* direction [Fig. 2(a)]. In the opposite channel, fluid is gained, which causes the pressure gradient to increase with *x*. This deviation makes it more difficult to keep Δ*P* constant. We therefore used this model to minimize the deviation in Δ*P* along the *x*-direction. To render the uniformity robust to experimental heterogeneities such as local variations in post height resulting from the microfabrication process, we set a low *x*-deviation criterion, at no more than the larger of 0.01 *μ*m s^−1^ or 1% of the average *y*-direction velocity.

We assumed steady and fully developed laminar flow in the fluid channels, where the Reynolds number is typically between 10^−4^ and 10^−2^. Their hydraulic resistance was calculated according to the Boussinesq equation for rectangular channels. Fluid flow in the porous gel region was calculated from Darcy’s law, an assumption justified by the spatial uniformity of the collagen I gel used here and the small size of the region at the fluid–gel interface where transitional flow occurs, i.e., 100 nm.^48^ All fluid flow across the gel was assumed perpendicular to the side channels, thus the passage between two opposite posts was modeled as a section separated from its neighbors [Fig. 2(b)]. The pressure and velocity distributions were calculated using a custom Matlab (MathWorks, USA) script that started with uniform and mean-valued advection and used the Boussinesq and Darcy equations sequentially to converge to a distribution that respected both physical laws. This is detailed in the supplementary material, Sec. 1. Collagen gel permeabilities at 2 mg ml^−1^ were reported over three orders of magnitude in the literature (0.1,^4^ 1,^49,50^ and 10 *μ*m^251^). Another study measured 4 mg ml^−1^ gels at 0.01 *μ*m.^27^ Therefore, we aimed for our chip to satisfy the advection deviation criterion over the widest range of permeabilities. Finally, in order to assess the robustness of our circuit to small differences in the supply flow rate, we simulated a pressuredriven setup where the supply lines upstream from the chip had different resistances. We represented them as 20 cm of 0.5 mm inner diameter tubing (such as WZ-06419-01, Cole Parmer, USA) in series with 20 cm of 0.065 mm inner diameter tubing (such as LVF-KTU-04, Elveflow, France) on the same side as the higher outlet resistance and 15–20 cm of the smaller tubing on the opposite side. Those dimensions are realistic in a microfluidic setup and cover the range of flow-rate differences that arise in pressure-driven circuits if the resistances of parallel paths differ.

### 3D computational model of flow and transport in the chip

The trapezoidal shape of the posts create variations in fluid velocity in the *y*-direction, which could locally skew concentration profiles at either side of the gel region. To quantify the effect of the gel section geometry on the post-processing of experimental gradients, we constructed an advection-diffusion model in Fluent (ANSYS, USA, parameters in Table I). The geometry of the gel region and adjacent fluid channels was meshed with tetrahedral elements using ANSYS’s ICEM CFD meshing tool. The mesh was refined by a factor 2 in a region extending 100 *μ*m on each side of the fluid–gel interfaces and a hexahedral core mesh was applied to the remainder of the domain. Results were also obtained from a custom 1D implicit upwinded finite-difference advection-diffusion solver in Matlab.

### Chemokine gradient analysis

**Fig. 3:**
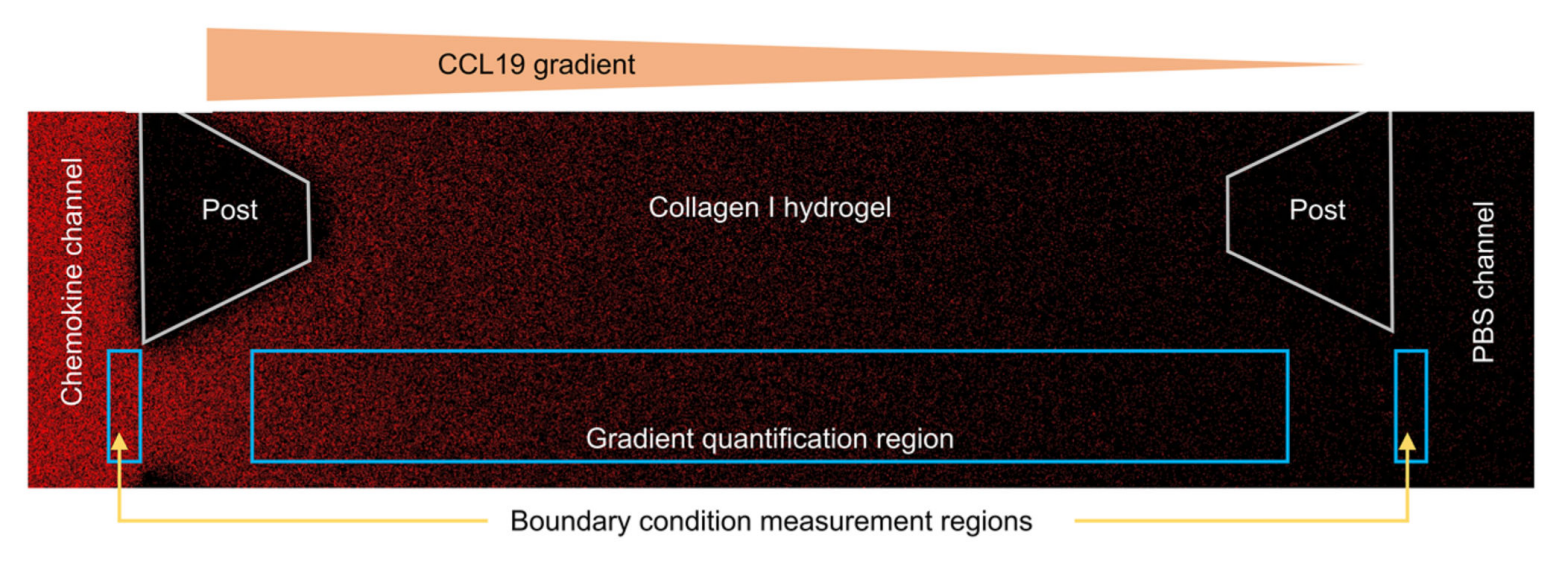
Confocal microscopy scan of steady-state AF647 CCL19 fluorescence with advection opposing diffusion. The chemokine source concentration is 10 nM and magnification is 10 ×.

A rectangular gradient quantification strip centered on the middle of a gel section was assigned to each image. Its exact dimensions were determined *ad hoc* from the position of the interfaces and the post edges on the brightfield images (Fig. 3). The first 100 *μ*m into the gel region were left out to reduce the effects of the non-planarity of the fluid–gel interfaces and the possible gel compression at the interfaces. A margin of at least 10 *μ*m was left between the rectangles and the post edges to eliminate shadow effects from the posts. The exact dimensions of the resulting observation window were used in subsequent analysis steps. The fluorescent intensity was averaged across quantification strips to reduce noise, which was justified by the realization of a rapid lateral and vertical diffusive equilibrium and the absence of flow in those directions. References for concentration boundary conditions were taken as the average intensities in 100 × 20 *μ*m^2^ rectangles in each fluid channel, adjacent to the base of the posts. This was chosen as a compromise between matching the fluid–gel interface and excluding the refractive effects that it could induce. The steady-state advection–diffusion equation was then solved using our custom finite-difference solver and the resulting concentrations were fitted to the observed steady-state concentrations using a normal likelihood estimator with standard deviation 1. The experimental Péclet number [Eq. (1)] was defined using the fitted velocity in the middle of the gel region *v*_mid_, which is the advection velocity for more than half the pathway along a gel section. The length scale, *L*, is the length of that section, which is 1.3 mm by chip design but was measured from the brightfield images to account for the exact position of fluid–gel interfaces. The diffusivity, *D*, is the one we measured as 129 *μ*m^2^ s^−1^ for CCL19 in the same experimental conditions,

(1) $$\text{Pe}=\frac{v_{\text{mid}}L}{D}.$$

## Results

### Tunable outlet resistances allow on-the-chip adjustment of gradient shape and direction

**Fig. 4:**
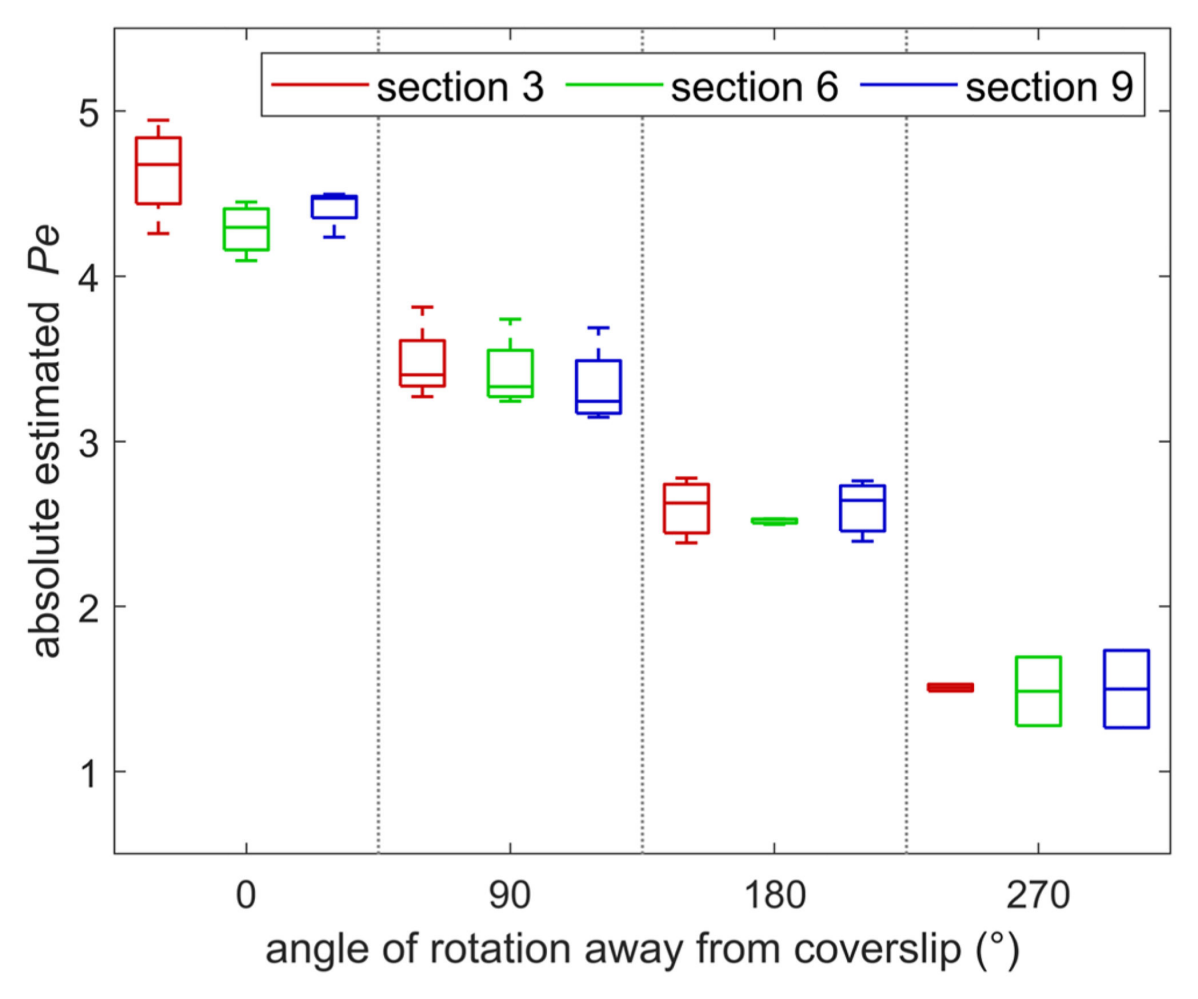
Absolute estimated Péclet number as a function of the angle of rotation of one screw away from its fully deflected position, at gel sections 3, 6, and 9 away from the inlet. Number of chips: *N =* 4 at 0 and 90°, *N =* 3 at 180°, and *N =* 2 at 270°. Repetitions were conducted from different chips.

**Fig. 5:**
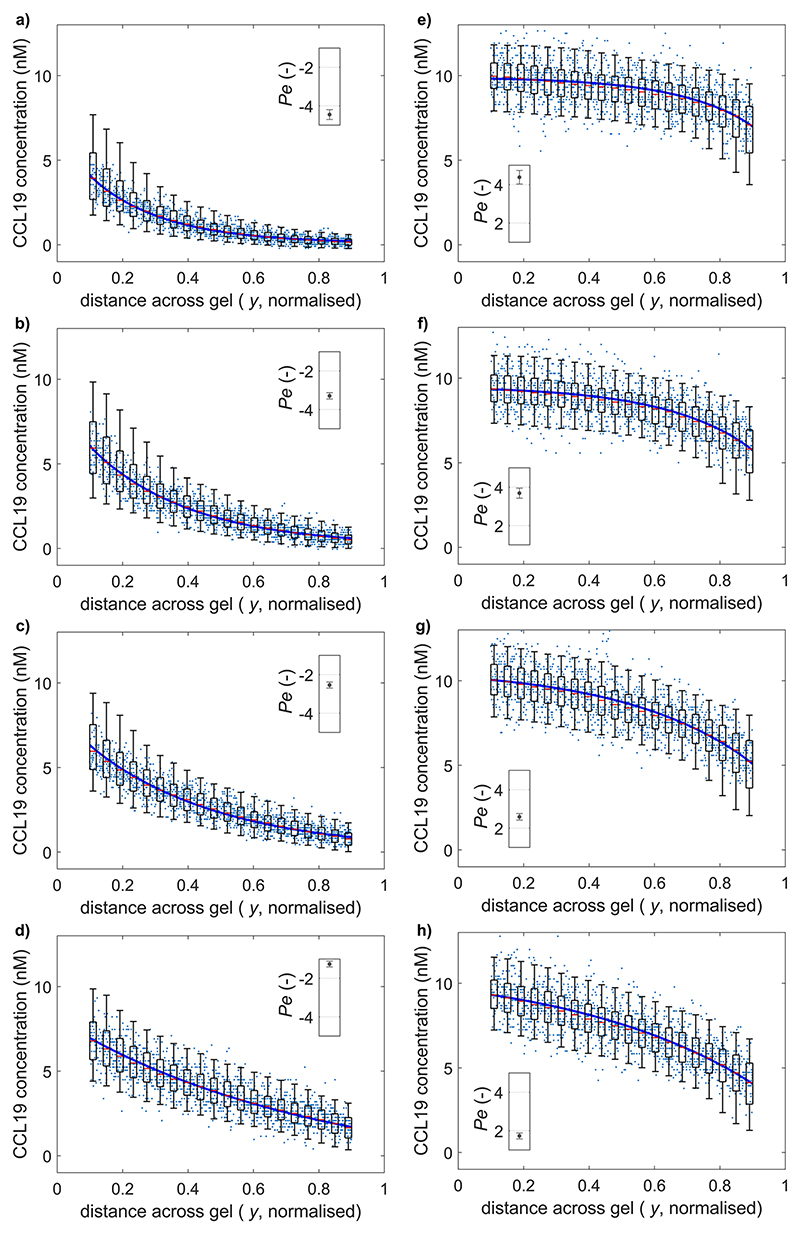
(a)–(d) Example steady-state CCL19 concentrations along a gel section with advection opposing diffusion. The sub-figures represent decreasing advection magnitude achieved by successive 90° rotations of one M1.4 screw away from its full deflection. (e)–(h) Steady-state concentrations with advection supporting diffusion and subfigures following the same progression. The boxes and whiskers each represent a Gaussian probability density estimate for 1/20^th^ of the gel section length. The centers of boxes represent 50% cumulative density, the box extremities 25% and 75%, and the whiskers 5% and 95%.

The displacement of the resistance membranes could be adjusted to control the magnitude of advection in the gel. This could be used to shape the formation of gradients across it with good repeatability. The variation in induced Pe by one fully deflected screw was less than 1 unit across four different chips, despite individual variations in the exact position of the screw above the channel and in the fluid channel height, which may affect the induced resistance (Fig. 4). Starting with the membranes on the source side open and one membrane on the buffer side completely closed, successive 90 counter-clockwise rotations of the screw on the buffer side allowed gradients of CCL19 with advection opposing diffusion at a Péclet number varying in the range [−5, −1] to be created [Figs. 5(a)–5(d)]. Conversely, advection enhancing diffusion was induced in the same range by starting with one source membrane completely closed and the buffer membranes open, and gradually opening the source membrane [Figs. 5(e)–5(h)]. These results imply that the advection resolution of the screws at the supply flow rate used here of 2 *μ*l min^−1^ is 0.1 *μ*m s^−1^/90°, which meets the precision requirement.

### Local advection velocity estimation

**Fig. 6:**
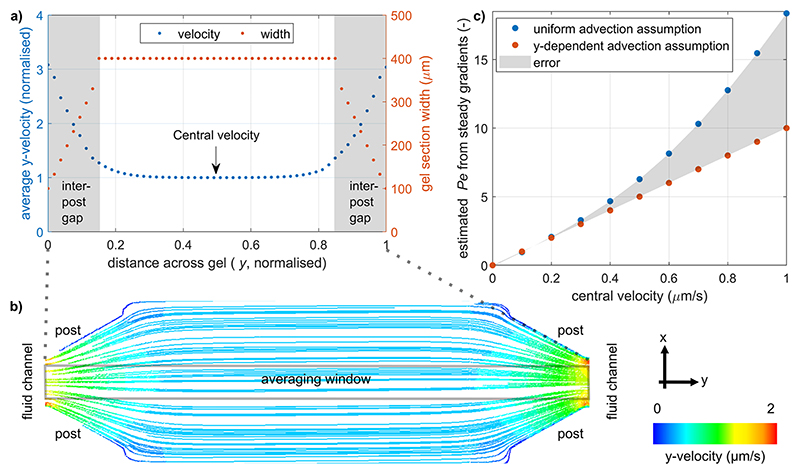
(a) Width of the gel section and average *y*-velocity between two opposing fluid–gel interfaces as computed in Fluent. (b) Particle streamlines in a gel section colored by *y*-velocity. This velocity varies little across the averaging window, justifying that the averaging does not introduce bias on the velocity estimated from experimental gradients. The *x*-velocity into the adjacent sections is 2.5 × 10^−3^ *μ*m s^−1^, i.e., at least two orders of magnitude lower than the *y*-velocity. (c) Improvement in Péclet number estimation from averaged steady-state concentration profiles by using the *y*-dependent shape of the velocity as opposed to assuming it constant. Gradients were computed in Matlab.

The conservation of fluid mass implies the acceleration of fluid flow at the locations where the path along a gel section narrows, i.e., between adjacent posts at each of its ends [Fig. 6(a)]. We fitted an analytical form to this velocity averaged across the width of an observation section [Fig. 6(b)] to improve the accuracy of the quantification of experimental gradients. Averaging was justified because the *y*-velocity varies along that dimension by at most 10%, close to the fluid–gel interfaces, and by 0.01% in the center of the gel region. We chose a piece-wise rational function, defined in Eq. (2), where *a*_*i*_, *b*_*i*_, *c*_*i*_, and *m* are fitting constants and *y*_*m*_ is the distance from the interfaces from which the velocity can be considered uniform,

(2) $$u(x)=\{\begin{array}{ll} \frac{a_{1}}{b_{1}y+c_{1}}, & 0\leq y<y_{m},\\ m, & y_{m}\leq y\leq 1-y_{m},\\ \frac{a_{2}}{b_{2}(1-y)+c_{2}}, & 1-y_{m}<y\leq 1. \end{array}$$

More detailed mathematical developments can be found in the supplementary material, Sec. 2. Using this position-dependent form of advection significantly improves its estimation for moderate and high velocities [Fig. 6(c)]. Assuming that the velocity is constant and fitting a simple exponential profile to the gradients would overestimate the Péclet number by 1 for a central Pe of 5, increasing to an overestimation by 8 for a central Pe of 10.

### Advection steadiness

**Fig. 7:**
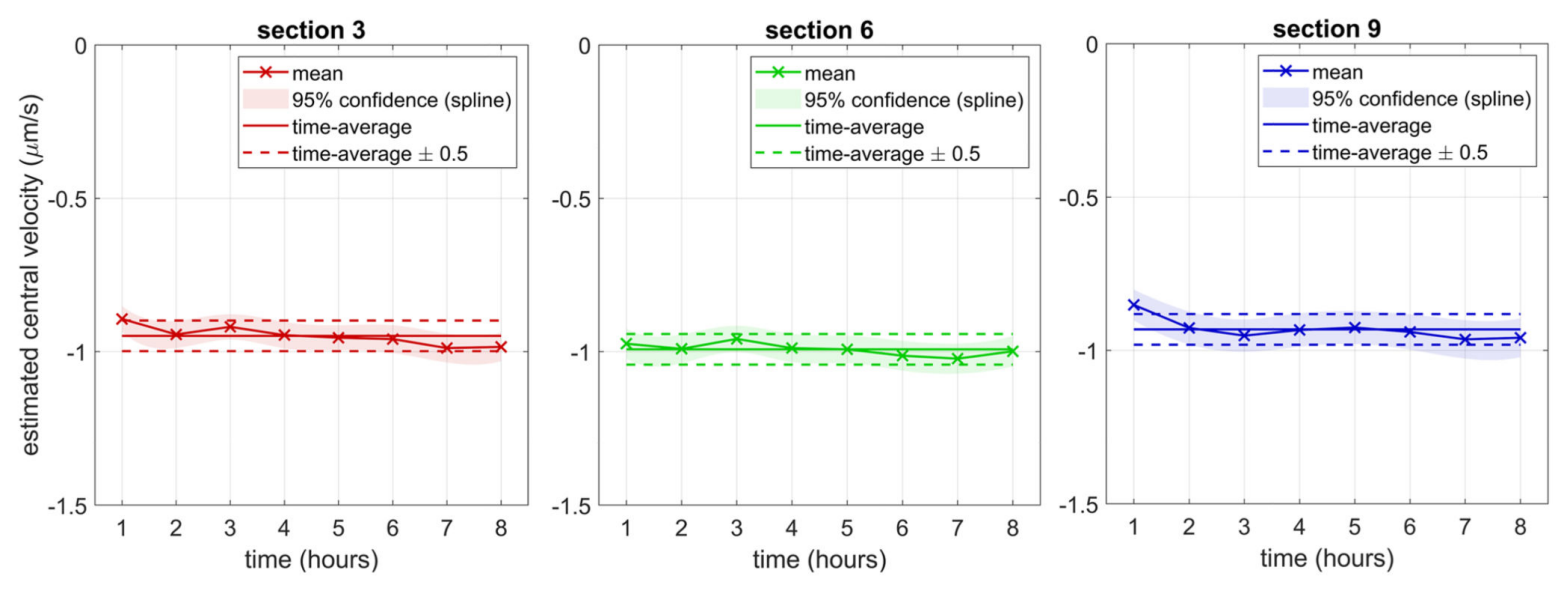
Evolution of the estimated central velocity over 8 h after the establishment of a steady-state gradient with advection opposing diffusion, at three different gel sections. Estimated velocities remain within a window of 0.1 *μ*m s^−1^ width at all sections and splined confidence intervals maintain a width of this magnitude at all time points, indicating stable advection.

Chemokine gradients generated with two fully deflected screws were steady for at least 8 h, insofar as the Péclet numbers estimated at 1-h intervals remained within a window of at most 1 Pe-unit width (Fig. 7). Their 95% confidence intervals also remained at widths of 1 Pe-unit, despite concentration profiles depending little on Pe when their advective component is this high, which reduces the precision of Pe estimations from concentration profiles in these contexts. Small variations may occur because of imaging-noise specific to the gel section and noise in the flow rate delivered by the syringe driver affecting flow in the entire chip. At Pe close to 10, these gradients are close to zero in the majority of the gel region and are, therefore, at the upper limit considered to be sensible to generate in this chip. The steadiness at those high Péclet numbers suggests steadiness across their range.

### Reducing flow resistance in gel-adjacent fluid channels increases advection uniformity

**Fig. 8:**
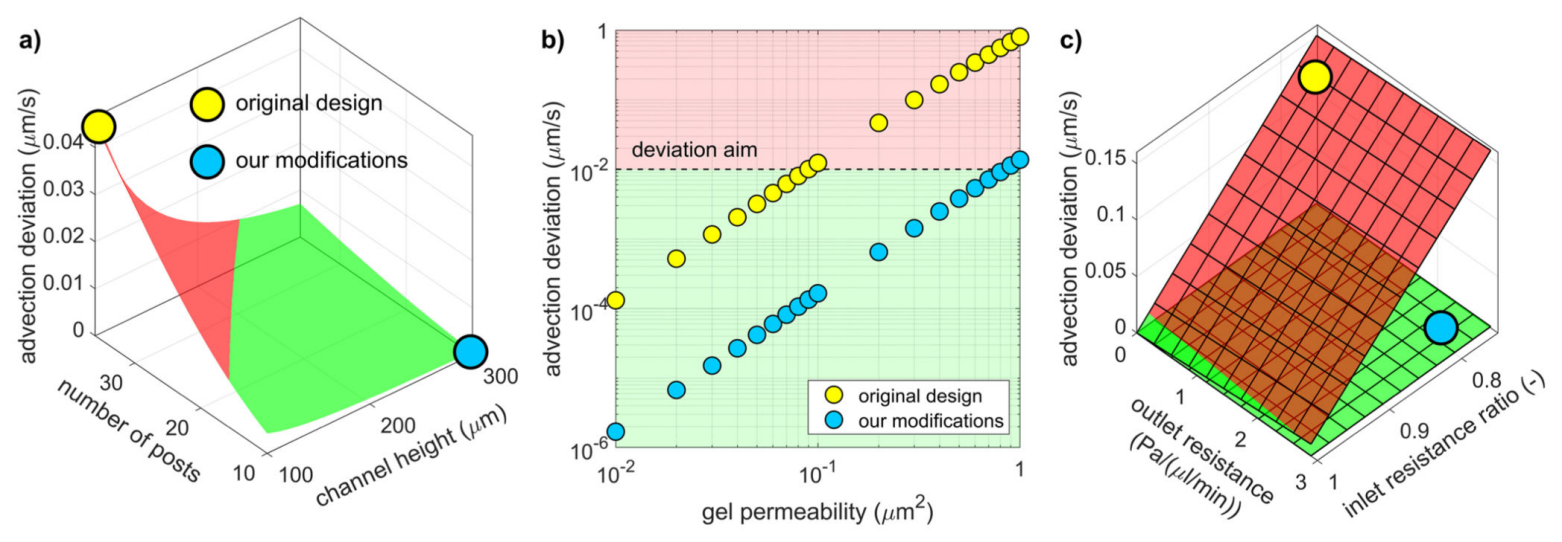
(a) Advection deviation against number of posts *N* and fluid channel height *h* for a fluid channel width of 500 *μ*m and equal supply flow rates. Example gel permeability of 0.2 *μ*m^2^. (b) Advection deviation against gel permeability for the original design and our modified version over the range of reported permeabilities of collagen gels. (c) Effect of differing supply flow rates on advection variation across gel sections for the original design^36^ and our adapted one, in the case of pressure-driven fluid supply. Reducing the resistances of the fluid channels provides robustness to those variations.

After the outlet resistances, the resistances of the fluid channels along the posts are a second source of pressure variation in the circuit. Reducing them decreased the sensitivity of the pressure in the fluid channels to the variation in the fluid-channel flow rate that results from mass exchange across the gel. This, in turn, reduced the deviation of advection along the gel region. This effect was independent of the resistance difference between the downstream sections. We applied it by reducing the length of the gel region, i.e., the number of posts, and increasing the hydraulic diameter of the fluid channels [Fig. 8(a)]. Our design modifications reduced the deviation of advection by two orders of magnitude over the range of reported collagen gel permeabilities and met the deviation aim of 0.1 *μ*m s^−1^ up to almost the upper limit of that range (1 *μ*m^2^) [Fig. 8(b)]. We considered variations in the supply flow rate as a third source of pressure difference between the two fluid channels. In the case of wide fluid channels where viscous losses are reduced, gel advection was tolerant to those variations and remained small, in contrast to the case of the original geometry where the fluid channel resistance was higher [Fig. 8(c)]. This tolerance increased with inlet paths of higher resistance, which can be designed by incorporating thin tubing into the supply lines. Nevertheless, we chose a pump-driven supply over a pressure-driven one for its simplicity of use, portability, and ability to maintain sterile conditions for future cell migration experiments. We subjected our syringe drivers to velocity measurements by fluorescence recovery after photobleaching (FRAP) in a straight microfluidic channel to verify that they delivered constant and accurate flow (data not shown). Variations in syringe driver calibrations and possible low-amplitude oscillatory modes in motor operation necessitated the use of a common two-channel driver. The design and operation choices resulting from the application of the lumped-parameter model are summarized in Tables II and III.

**Fig. 9:**
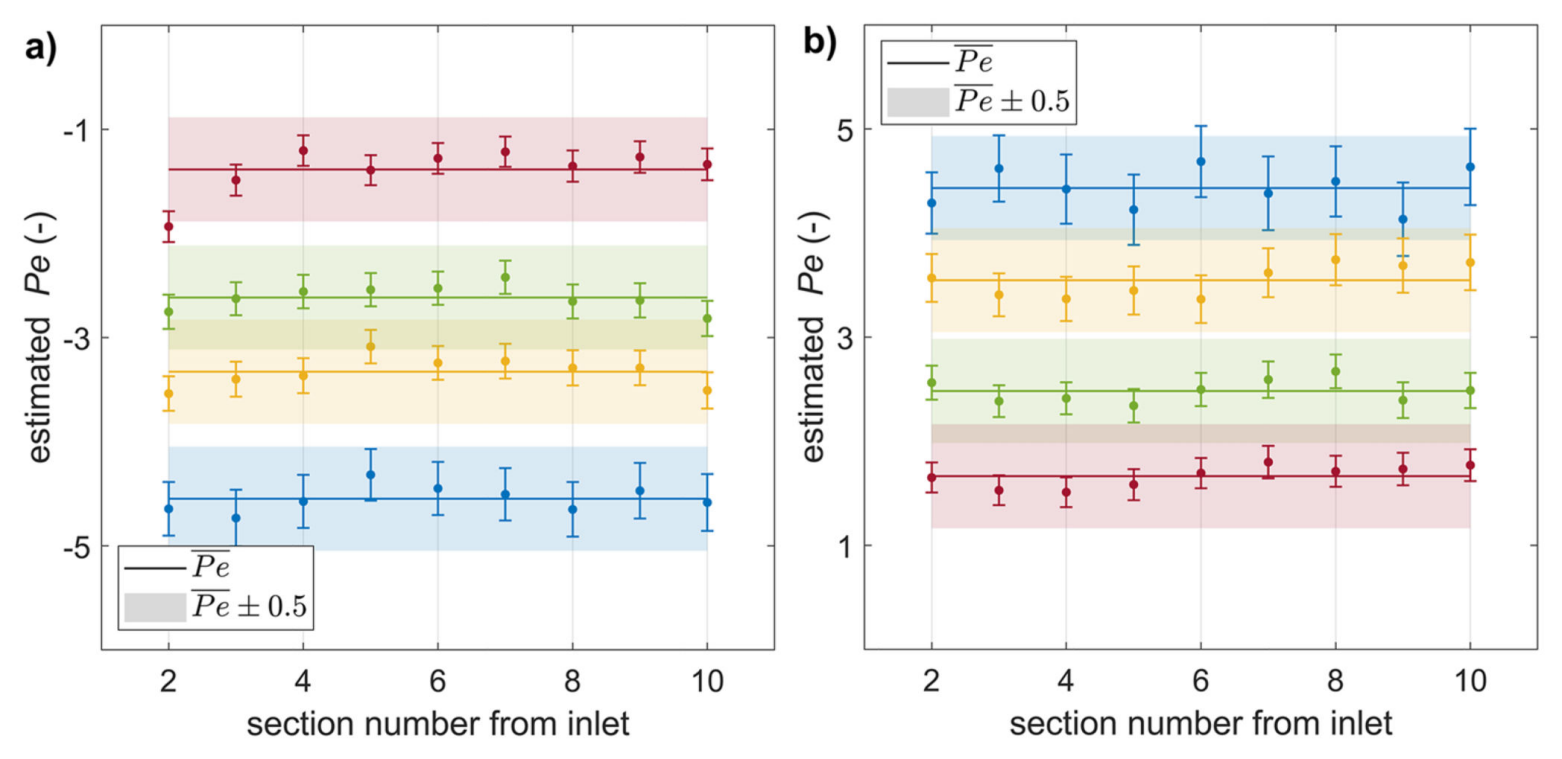
Estimated Péclet numbers from experimental steady-state CCL19 gradients against the position along the chip, with gel section numbers increasing in the direction of the outlet. Successive 90° screw rotations in the same chip. (a): advection opposing diffusion and (b): advection enhancing diffusion. The variations can be due to experimental inhomogeneities and imaging-noise and do not indicate that the chip geometry could prevent uniform advection.

Experimentally, the estimated Péclet numbers deviated by less than 0.5 units from their average across gel sections (Fig. 9). No pattern of deviation from the average could be consistently identified across experiments. The deviation is larger than the design criterion of 0.01 *μ*m s^−1^, or 0.1 Péclet units, which can be explained by imaging-noise, by errors arising from the compromise made in the choice of domain for the measurement of boundary conditions, and by fluid–gel interfaces whose variations in position and shape can affect the resistance of the gel section they delimit. However, it is within the precision requirement of controlling advection to 0.1 *μ*m s^−1^, or 1 Péclet unit, for CCL19.

## Discussion

### *In vivo* fidelity

Four characteristics of chemokine gradients should be considered prior to reproducing them with physiologic fidelity: advection velocity, source concentration, distance between source and buffer, i.e., gradient range, and angle between the directions of diffusion and advection. Here, our priority was advection velocity, as it was absent from previous *in vitro* studies. *In vivo*, it varies among tissue types and depends on local physiologic and pathologic conditions.^52^ It was measured at 0.1–1 *μ*m s^−1^ in homeostatic conditions in rabbit ears,^34^ and up to fivefold increases were reported upon contact inflammation in mice.^41,53,54^ In one of the very few human studies, IF in brain tumors was consistently of the order of 10 *μ*m s^−1^ and could increase up to 50 *μ*m s^−1^ in patients with metastatic cancer.^55^ The experimental Péclet numbers measured here for CCL19 correspond to homeostatic velocities. They could be increased to the higher velocities associated with inflammation by implementing device design changes such as narrowing the gel region or adding more screws in series downstream of the gel. Another of our objectives was to observe gradients at physiologic source concentrations. The concentration of chemokine might have an effect on cellular response through the degree of receptor occupancy, in addition to the differential receptor occupancy that gradients induce. We imaged gradients from chemokine sources of 10 nM, which corresponds to the upper range at which CCL19 was reported in mouse lymph nodes.^46^ While this concentration was at the limit of detectability of the confocal microscope we used, it is in the lower range of concentration measurements for other chemokines. This indicates that the gradients of many of them could also be quantified at physiologic concentrations. It also suggests that the quantification of gradients in environments where chemokines are subject to ECM-binding and removal could be possible by direct observation. A study using a higher-concentration dextran surrogate would have left this question unanswered. Regarding the gradient range, the distance of 1.3 mm between the source and buffer in our chip is an order of magnitude larger than the few *ex vivo* gradient observations reported in mice lymph nodes^56^ and subcutaneous tissue,^57^ but as there has been no study of that range across organs or across species, we did not modify that dimension, nor do we recommend a range for it. Keeping it thus allows the observation of many cells over a relatively compact region, short in the direction of supply flow, which reduces the deviation of advection along it [Fig. 8(a)]. Studies of gradients in specific contexts may modify the source–buffer distance in order to suit the physiologic environment that is being reproduced and adapt the advection generation parameters accordingly. Finally, the relative angle of diffusion and advection directions may take any value *in vivo*, for example, in the case of autologous gradients. The device presented here is aimed primarily at the study of long-range signals and migration, e.g., homeostatic or inflammatory migration to subcutaneous lymphatics, migration toward tumors, or toward inflamed large blood vessels from their perivascular tissue. In these contexts, the directions of diffusion and advection are parallel, i.e., radially away from endothelia or tumors. This technique complements laminar flow patterning techniques, where gradients perpendicular to fluid flow can be generated with great flexibility.^11,19^ Still, varying the angle between diffusion and advection on-chip remains a challenge.

### Design and operational choices

Torque-actuated valves provide superior pressure-control precision and stability than setups based on differences in liquid heights. Typical setups driving IF with pressure heads have a resolution of 10 Pa, notably the ones that were used to impose flow on top of growth factor gradients.^25,26^ This is too coarse for the widely used collagen I gels employed in this study and could be mitigated only partly by modifying the gel region geometry (see the supplementary material, Fig. 1). Besides, these height differences decrease exponentially with time. Even though the decay constant can be increased by using wider reservoirs and a resistive circuit between them and the observation region,^58^ this would only delay the equilibrium of liquid heights. Therefore, liquid-height setups are only able to provide a constant average velocity or one that is superior to a threshold for long periods. They require precise and continual adjustment of liquid levels, in particular, if instantaneous IF, and not just its average value, is an experimental goal. Screw-induced resistances, by contrast, can be maintained indefinitely without external power while keeping the setup compact. Their adjustment after their initial calibration is nearly instantaneous. The uncertainty on the resistance value, which stems from variations in the screw position over the fluid channel and in the fluid channel height, was not detrimental to the repeatability of our experiments as Pe remained in windows of 1 Pe-unit width across resistances, within chips, and across chips. The resistance membranes had a resolution of 10 Pa per 90° or lower at the flow rate of 2 *μ*l min^−1^ used in the experiments presented here, which is comparable to the resolution of around 1 mm H_2_O achieved by pressure-head systems. However, contrary to pressure-head systems, there is scope to make the resolution of resistance membranes finer: first, by minimizing the supply flow rate within the constraints of keeping boundary concentrations constant and advection deviation low; second, by exploring the feasibility of having membranes in parallel channels on each outlet branch.

The deflection of membranes by screws enabled a device with a low footprint and an operation of membranes that was both direct and with tolerance on fabrication variations. The rotation of the screw alone determined the deflection depth of the membrane, and the pressure applied to the top of the membrane adapted naturally. It could thus be applied with some tolerance on membrane thickness. This enabled us to fabricate membranes through the simple and cheap means of inserting 3D-printed racks into uncured PDMS. This produced a two-layer chip in one block, with neither a need for careful alignment nor a risk of delamination. The subsequent operation of the chips was straightforward with the only remaining task being to position the screws above their indents and calibrate their lowest position on the microscope stage. In contrast, the widely used method of deflecting membranes onto a microfluidic channel by pneumatic actuation requires additional tubing and an external control setup. Whether the latter is manual or automatic, it increases complexity and bulk. We also found pneumatic actuation to be prone to causing bubbles due to air flow from the compressed air line into the chip across the semi-permeable PDMS membranes. Torque-actuated deflection engenders no such difficulties.

### Limitations and future work

The pressure drop induced by the deflection of objects onto fluid channels is limited by geometry in two ways. First, there exists a minimal non-zero channel cross-section resulting from the channel being slightly wider than the actuators. Second, the fluid channel length available to deflect objects is constrained by the size of the chip. The value of maximum advection resulting from this pressuredrop limit depends on the resistance of the upstream gel region. Simple modifications such as increasing the length over which an object is deflected or reducing the width of the gel region can render our setup suitable to gels whose permeability is more than one order of magnitude lower than that of collagen I. Such gels would still be more permeable than many tissues.^59^ Space constraints on the chip and the fact that the permeabilities of hydrogels can span several orders of magnitude represent a challenge to increasing the range of situations to which our method of resistance-driven advection is applicable. Therefore, future designs that do not impose an upper limit on advection could require a different method from the deflection of a membrane and might need to compromise on some of the portability and simplicity of our setup.

The linear relationship observed between the angle of rotation of the resistance screws and the advection induced in the gel is not predicted by the theoretical relationship between the deflection of an elastic membrane and the cross-section of a rigid channel underneath. Our hypothesis is that the rate of decrease in advection with membrane relaxation is slowed down because of a combination of channel wall deformation and membrane hysteresis. Each membrane was deflected down to the coverslip surface before being used to generate advection, which gave it a history of strain. Others who designed torque-actuated valves over deep channels, like in the chip presented here, also observed hysteresis as well as a linear decrease in the flow rate under the valve with its increasing deflection depth.^44^ Further work including pressure drop measurements and numerical modeling of the membranes will seek to quantify this effect in our circuit. It will aim to improve the understanding of the relationship between the degree of torque-valve actuation and the advection it induces, and more broadly to increase the scalability of this method.

The flow rate supplying chemokine and PBS in the side channels affects the precision and uniformity of the gradients in three ways. First, the maintenance of constant boundary concentrations on each side of the gel requires a high supply and removal velocity. We determined empirically that 100 *μ*m s^−1^ was necessary for the fluorescent intensity in a channel previously filled with PBS to stabilize within 10 min of switching the fluid supply. This velocity leads to an inefficient use of chemokine as much of the chemokine flows past the observation region. Second, in highly advective conditions, the uniformity of boundary concentrations along the *x*-direction is reduced by the formation of a diffusive boundary layer in the channel receiving fluid from the gel region. This is due to the alternation between wall conditions at the post bases and the supply of fluid mass across the fluid–gel interfaces. This boundary layer widens as more mass crosses the interfaces, i.e., as the Péclet number increases and as the fluid progresses toward the outlet in the side channels, encountering more interfaces along the way. This leads the boundary concentrations in the channel receiving fluid to increase along *x* at positive Pe and decrease at negative Pe. Thus, the gradients with a large advective component become near zero along the entirety of the gel sections closer to the outlet. The uniformity of gradients across those sections is reduced and the estimation of their Péclet number is difficult. Our baseline flow rate of 2 *μ*l min^−1^ was high enough to prevent this effect at Pe up to 5 but was noticeable at higher Pe (see the supplementary material, Fig. 2). Third, the pressure drop induced by the deflection of the screws could be decreased by reducing the supply flow rate. This would increase the precision of the advection control, which could be useful in studies that require advection to be established across a narrower gel region. Therefore, varying the supply flow rate could represent an opportunity to establish controlled gradients in a wider variety of contexts, including ones of high advection and of high pressure-differential precision.

### Perspectives

This microfluidic chip combined fine flow control at a precision of the order of 0.1 *μ*m s^−1^ across a three-dimensional gel with the generation of stable chemokine gradients. This degree of finetuning allows the simulation of the full variety of advective–diffusive transport situations occurring *in vivo*. Experiments involving local and precise flow modifications that are only possible *in vitro* will help to elucidate the crucial but not widely understood role of fluid flow in shaping functional chemokine gradients *in vivo*. In particular, the chip we presented allows future work to disambiguate the effects of skewed chemokine gradients from the effects of IF itself. For this, cell migration would be quantified in response to advective–diffusive gradients against controls with flow only. It would also be possible to generate skewed solid-phase gradients in a gel containing chemokine-binding ECM molecules before stopping the advection that skewed them. Furthermore, our ability to quantify the formation of gradients at physiologic concentrations enables the future simultaneous observation of gradient evolution and cell migration.

Given experimental limitations on chemokine gradient quantification, *in silico* and *in vitro* techniques have the potential to reveal additional, useful information on chemokine transport. Chemokine gradients have, indeed, not yet been observed or quantified *in vivo*. The most common *ex vivo* detection method, immunostaining with a chemokine antibody, requires time-consuming tissue fixation, which also removes all unbound chemokines.^56,57,60,61^ This process can drastically change the chemokine concentration field from its dynamic *in vivo* state and does not afford insight into the roles of the multiple biophysical and biochemical processes that shape the observed gradients. Diffusion-reaction mathematical models^62–64^ have shed light onto the importance of ECM binding, cell clearance, and spatiotemporal distribution of chemokine production in shaping gradients. Including advection in transport models has shown that cells greatly increased autologous gradients in either high-IF situations or in response to heavy molecules,^23^ thereby increasing their probability to migrate.^63^ Under high IF, tumor cells producing their own chemokines migrated upstream even in the absence of an external gradient.^3^ By incorporating the above phenomena at the scale and geometry of a lymph node, we were able, in previous work, to refine *ex vivo* findings by Ulvmar *et al*.^56^ by exposing the combined effects of lymph flow and binding to the atypical chemokine receptor ACKR4 on the shape of CCL21 gradients across the node paracortex.^65^ Quantitative and context-specific information on chemokine gradient formation and maintenance could inform some of their *in vivo* characteristics such as stability, range, and potency, generate hypotheses for *in vivo* experiments, and improve the prediction of the efficacy of therapeutic strategies targeting chemokine gradients. The simultaneous fine controls of fluid flow and diffusive transport in microfluidic models represent a step in that direction.

## Supplementary Material

The supplementary material provides details of mathematical developments of the lumped-parameter model and the gradient quantification and figures supporting the discussion of pressure-control resolution and supply flow-rate requirements.

Supplementary Material

## Figures and Tables

**Table I:** Boundary conditions used in the 3D computational fluid simulations. S and B represent the source and buffer as in Fig. 2(b).

Boundary	Laminar flow solver conditions			Advection-diffusion solver conditions	
	Type	Value		Type	Value
Inlet S	Flow rate	2 *μ*l min^−1^		Dirichlet	*C* = 1
Inlet B	Flow rate	2 *μ*l min^−1^		Dirichlet	*C* = 0
Outlet S	Pressure	0		Neumann	*D*∇*C* ⋅ **n** = 0
Outlet B	Pressure	0–10 Pa		Dirichlet	*C* = 0

**Table II:** Effects of experimental design parameters on advection uniformity and magnitude criteria. *U* = advection deviation from uniformity; $\bar{v}$ = average advection velocity across gel sections; other symbols as in Fig. 2(b).

Design parameter	Symbol	Primary effect	Secondary effect
Number of posts	*N*	*N* ↘ ⇒ *U* ↘	None
Fluid channel resistance	*R^c^*	*R^c^* ↘ ⇒ *U* ↘	None
Gel permeability	*k^g^*	*k^g^* ↘ ⇒ *v̄* ↘	*k^g^* ↘ ⇒ *U* ↘
Gel region width	*w* ^g^	*w^g^* ↘ ⇒ *v̄* ↗	*w*^g^ ↘ ⇒ *U* ↗
Outlet resistance difference	Δ*R_out_*	Δ*R^out^* ↘ ⇒ *v̄* ↗	Negligible
Supply flow rate	*Q_in_*	*Q^in^* ↘ ⇒ *v̄* ↗	Negligible
External pressure difference	Δ*P_out_*	Δ*P^out^* ↘ ⇒ *v̄* ↗	Negligible

**Table III:** Indirect effects of design parameters on experimental characteristics and chip operation. BC boundary concentrations; other symbols as defined in Table II.

Symbol	Other benefits of primary effect	Negative consequences of primary effect	Design outcome
*N*	Easier gel injection less variation in BC	Smaller observation area for cells	10
*R^c^*	Δ*R_out_* adjustment easier	Stable BC need higher *Q_in_*	*h^c^* = 300 *μ*m, *w^c^* = 1 mm
*k^g^*	May be closer to tissue permeability	Δ*R_out_* needs adjusting to maintain the advection range	Guided by experiment goals
*w^g^*	May be more physiologic	Shorter distance to track cells	1.3 mm (no modification)
Δ*R_out_*	None	None	Design choice
*Q_in_*	Faster establishment of stable BC	Chemokine supply less efficient	2 *μ*l min^−1^ baseline
Δ*P_out_*	None	$\bar{v}$ not constant	No external heads

## Data Availability

The data that support the findings of this study are available from the corresponding author upon reasonable request.
