# Supplementary material for: Generation of stable advective-diffusive chemokine gradients in a three-dimensional hydrogel
Source: AIP Adv. Author manuscript; Available in PMC 2025 Jul 30. (PMC7617951; doi:10.1063/5.0064947)
Supplement: Supplementary Material [file EMS207213-supplement-Supplementary_Material.zip › Supplementary_online_material.pdf]

Generation of stable advective-diffusive chemokine gradients in a three-dimensional hydrogel: Supplementary Online Material

Willy V. Bonneuil,[1] Daniel J. Watson,[1] Jennifer Frattolin,[1] Matthew J. Russell,[2] Francesca Fasanella Masci,[3] Mikaila Bandara,[3] Bindi S. Brook,[2] Robert J. B. Nibbs,[3] and James E. Moore Jr[1]
[1)] *Department of Bioengineering, Imperial College London*
[2)] *School of Mathematical Sciences, University of Nottingham*
[3)] *School of Life Sciences, University of Glasgow*

I. LUMPED-PARAMETER CALCULATION OF PRESSURES AND FLOW RATES IN THE CHIP

The pressure at the post gap closest to the outlet is known by

$$P_1^S = P_{\text{atm}} + Q_{\text{out}}^S \; R_{\text{out}}^S \tag{1}$$

Since the distribution (Q^g) is not previously known, the system is under-defined. The ideal uniform pressure difference across the gel region is due to the outlet resistance difference only and would be $Q_{\text{in}}^S \, (R_{\text{out}}^S - R_{\text{out}}^B)$. Let

$$\overline{Q^g} = \frac{1}{N+1} Q_{\text{in}}^S \, \frac{R_{\text{out}}^S - R_{\text{out}}^B}{R^g} \tag{2}$$

be the corresponding uniform ideal cross-gel flow rate. The real flow rate can be decomposed into

$$Q_n^g = \overline{Q^g} + Q_n^{g\,\prime} \tag{3}$$

with $\sum_n Q_n^{g\,\prime} = 0$. The following algorithm converges to a distribution that respects both Boussinesq and Darcy laws.

- $(Q^g)(0) \leftarrow \overline{Q^g}$, $i \leftarrow 1$

do

- $(Q^S)(i)$ and $(Q^B)(i)$ by mass conservation
- $P_{N+1}^S(i)$ and $P_{N+1}^B(i)$ by Boussinesq's law in the outlet section
- $(P^S)(i)$ and $(P^B)(i)$ for i in $[\![1, N]\!]$ by Boussinesq's law along the posts
- $(Q^g)(i)$ by Darcy's law across the gel
- $i \leftarrow i + 1$

while $||(Q^g)(i) - (Q^g)(i-1)||_2 \geqslant \varepsilon$

This algorithm takes between 5 and 7 iterations to converge for $\varepsilon = 10^{-16}$, depending on the input parameters.

II. SOLUTION OF THE STEADY TRANSPORT EQUATION COUPLED WITH SPACE-DEPENDENT VELOCITY IN THE GEL REGION

The steady concentration of chemokine in the hydrogel domain is governed by

$$D\,\nabla^2 C - \mathbf{u}\cdot\nabla C = 0, \tag{4}$$

where $C(x, y, z)$ is the chemokine concentration and D the chemokine diffusivity in the hydrogel. Assuming that the chemokine is in diffusive equilibrium in the x- and z-directions, and that no-flux conditions are applied at the chip walls (x and z boundaries), the problem reduces to

$$D\,C'' - v(y)\,C'(y) = 0, \tag{5a}$$
$$C(y_{\text{S}}) = C_{\text{S}}, \tag{5b}$$
$$C(y_{\text{B}}) = C_{\text{B}}, \tag{5c}$$

where $C_{\text{S}}, C_{\text{B}}$ are the experimentally measured chemokine concentrations at opposing fluid-hydrogel interfaces. S and B represent source and buffer, respectively. $v(y)$ is the y-component of the velocity across the hydrogel. We introduce dimensionless variables (with hats) by defining

$$y = L\widehat{y} + y_0 \tag{6a}$$
$$C(y) = (C_0 - C_{n-1})\widehat{C}(\widehat{y}) + C_{n-1}, \tag{6b}$$
$$u(\hat{y}) = U\widehat{u}(\widehat{y}), \tag{6c}$$

where $L = y_{n-1} - y_0$, $U = u(y_0)$. We also define a baseline Péclet number $\text{Pe}_0 = UL/D$. Then

$$\widehat{C}''(\widehat{y}) - \text{Pe}_0\,\widehat{u}(\widehat{y})\widehat{C}'(\widehat{y}) = 0, \tag{7a}$$
$$\widehat{C}(0) = 1, \tag{7b}$$
$$\widehat{C}(1) = 0. \tag{7c}$$

From here, we drop the hats for ease of reading. Variables remain dimensionless. Equation (7a) has the general solution

$$C(y) = A\int_0^y \exp\left(\int_0^{s_2} \text{Pe}_0\,u(s_1)\,\mathrm{d}s_1\right)\mathrm{d}s_2 + B, \tag{8}$$

in which we haven't yet applied the boundary conditions. We seek a closed-form expression for the concentration while incorporating the velocity field. So, we write the velocity as a piecewise function that we fit to the velocity data. The form of the velocity is chosen carefully to ensure that the integrals in the general solution can be performed exactly.
The piecewise velocity function that we use is

$$u(y) = \begin{cases} \frac{a_1}{b_1 y + c_1}, & 0 \le y < y_m, \\ m, & y_m \le y \le 1 - y_m, \\ \frac{a_2}{b_2(1-y)+c_2}, & (1-y_m) < y \le 1, \end{cases} \tag{9}$$

where the a_i, b_i, c_i and m are constants determined by fitting this function to the velocity data, and y_m is location where the fitted velocity switches to a constant in the central part of the hydrogel. Plugging (9) into (7), we have to solve for the concentration in each of the 3 regions and enforce the continuity of the concentration and its gradient between regions.

For $0 \le y < y_m$, we find

$$C_{\text{left}}(y) = A_{\text{left}} \frac{c_1}{\text{Pe}_0\, a_1 + b_1} \left[\left(1 + \frac{b_1}{c_1} y\right)^{1 + \frac{\text{Pe}_0\, a_1}{b_1}} - 1 \right] + B_{\text{left}}, \tag{10}$$

where A_{left} and B_{left} are constants. Imposing the inlet boundary condition fixes $B_{\text{left}} = 1$, giving

$$C_{\text{left}}(y) = 1 + A_{\text{left}} \frac{c_1}{\text{Pe}_0\, a_1 + b_1} \left[\left(1 + \frac{b_1}{c_1} y\right)^{1 + \frac{\text{Pe}_0\, a_1}{b_1}} - 1 \right], \tag{11}$$

For $y_m \le y \le (1 - y_m)$, we find

$$C_{\text{mid}}(y) = A_{\text{mid}} \frac{1}{\text{Pe}_0\, m} \left(e^{\text{Pe}_0\, m y} - 1\right) + B_{\text{mid}}, \tag{12}$$

where A_{mid} and B_{mid} are constants.
For $(1 - y_m) < y \le 1$, we find

$$C_{\text{right}}(y) = A_{\text{right}} \frac{c_2}{\text{Pe}_0\, a_2 - b_2} \left(1 + \frac{b_2}{c_2}\right)^{\frac{\text{Pe}_0\, a_2}{b_2}} \left[\left(1 + \frac{b_2}{c_2}(1 - y)\right)^{1 - \frac{\text{Pe}_0\, a_2}{b_2}} - 1 \right] + B_{\text{right}}, \tag{13}$$

where A_{left} and B_{left} are constants. Imposing the outlet boundary condition fixes $B_{\text{right}} = 0$, giving

$$C_{\text{right}}(y) = A_{\text{right}} \frac{c_2}{\text{Pe}_0\, a_2 - b_2} \left(1 + \frac{b_2}{c_2}\right)^{\frac{\text{Pe}_0\, a_2}{b_2}} \left[\left(1 + \frac{b_2}{c_2}(1 - y)\right)^{1 - \frac{\text{Pe}_0\, a_2}{b_2}} - 1 \right]. \tag{14}$$

It now remains to find the coefficients A_{left}, A_{right}, A_{mid} and B_{mid} by imposing continuity of the concentration and its gradient. This gives the 4×4 linear system

$$\begin{pmatrix} \frac{c_1 \left[\left(1 + \frac{b_1 x_m}{c_1}\right)^{1 + \frac{\text{Pe}_0\, a_1}{b_1}} - 1\right]}{\text{Pe}_0\, a_1 + b_1} & 0 & \frac{1 - e^{\text{Pe}_0\, m x_m}}{\text{Pe}_0\, m} & -1 \\ 0 & \frac{c_2 \left(1 + \frac{b_2}{c_2}\right)^{\frac{\text{Pe}_0\, a_2}{b_2}} \left[\left(1 + \frac{b_2 x_m}{c_2}\right)^{1 - \frac{\text{Pe}_0\, a_2}{b_2}} - 1\right]}{\text{Pe}_0\, a_2 - b_2} & \frac{1 - e^{\text{Pe}_0\, m(1 - x_m)}}{\text{Pe}_0\, m} & -1 \\ \left(1 + \frac{b_1 x_m}{c_1}\right)^{\frac{\text{Pe}_0\, a_1}{b_1}} & 0 & -e^{\text{Pe}_0\, m x_m} & 0 \\ 0 & \left(1 + \frac{b_2}{c_2}\right)^{\frac{\text{Pe}_0\, a_2}{b_2}} \left(1 + \frac{b_2 x_m}{c_2}\right)^{-\frac{\text{Pe}_0\, a_2}{b_2}} & -e^{\text{Pe}_0\, m(1 - x_m)} & 0 \end{pmatrix} \begin{pmatrix} A_{\text{left}} \\ A_{\text{right}} \\ A_{\text{mid}} \\ B_{\text{mid}} \end{pmatrix} = \begin{pmatrix} -1 \\ 0 \\ 0 \\ 0 \end{pmatrix}, \tag{15}$$

which can be solved exactly (but the expressions are unwieldy), so instead we solve numerically for a given set of parameters.

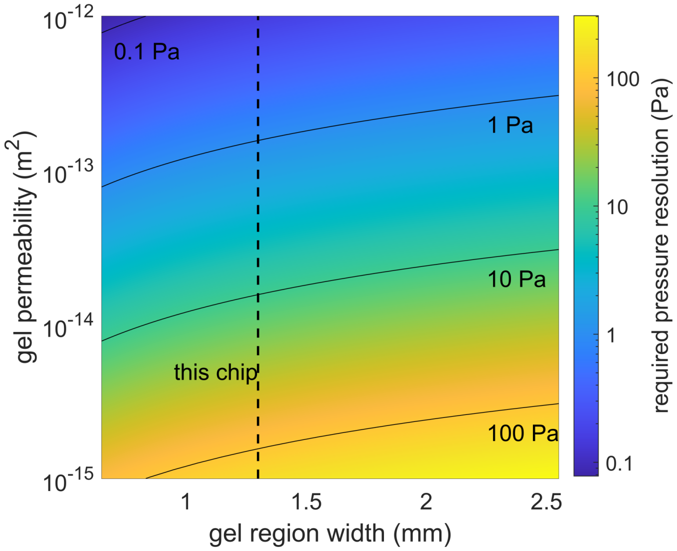

FIG. S1. Necessary resolution of the system controlling the pressure on both sides of the gel region to achieve a precision of 0.1 $\mu m\,s^{-1}$ in the induced advection. Resolution obtained by application of Darcy's law across the bulk of the gel region: $\Delta P = \mu w v/k$ with μ the fluid viscosity taken as that of water at 20°C, w the width of the gel region, v the target velocity of 0.1 $\mu m\,s^{-1}$, and k the gel permeability.

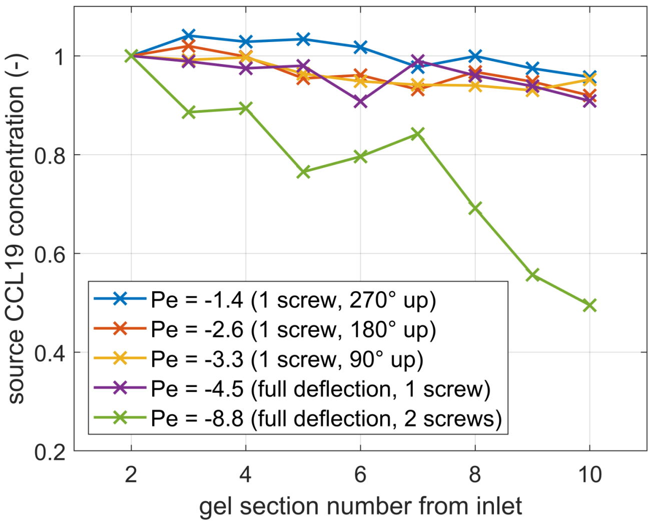

FIG. S2. Boundary concentration of CCL19 at the interface between the gel region and the fluid channel receiving mass through the gel, i.e. in the direction of advective transport across the gel. Concentrations expressed in relative value to the concentration at the observation section closest to the inlet. As the observation window moved towards the outlet, they decreased by less than 10% from their reference value for advection generated by up to one fully deflected screw, i.e. an absolute Péclet number within [0,5] for CCL19. At stronger advection magnitudes, the interface concentrations decreased by up to half of their reference value.
